# Quality of life in patients with psoriasis

**DOI:** 10.1186/1477-7525-4-35

**Published:** 2006-06-06

**Authors:** Monali J Bhosle, Amit Kulkarni, Steven R Feldman, Rajesh Balkrishnan

**Affiliations:** 1 Ohio State University College of Pharmacy and School of Public Health, 500 W. 12th Avenue, Columbus, OH 43210, USA; 2 Center for Dermatology Research, Department of Dermatology, Wake Forest University School of Medicine, Medical Center Boulevard, Winston-Salem, North Carolina, 27157-1071, USA

## Abstract

Psoriasis is one of the prevalent skin conditions in the United States. This chronic condition has a significant negative impact on patients' quality of life. Psoriasis has been linked to the depression and suicidal tendencies in the patients. The costs associated with decrements in quality of life, lost productivity, and work absenteeism may be enormous, increasing overall costs associated with the disease management. This review attempts to outline different quality of life measures available for psoriasis and describes their use in studies examining patient reported outcomes associated with pharmacological interventions for psoriasis. Factors associated with quality of life in psoriasis patients are described. It further describes physician's role in the psoriasis management to improve patients' overall well-being.

## Review

### Psoriasis: a growing problem

Psoriasis affects approximately 2% of the world's population, with men and women being equally affected [1]. In the United States (US) about 250,000 new cases of psoriasis are observed annually, affecting almost 2.2% of the US population [2,3]. Psoriasis accounted for nearly 2.25 million visits to ambulatory care centers during 1996 in the US [4].

Psoriasis is a serious condition strongly affecting the way a person sees himself and the way he is seen by others. It has tremendous economic and financial ramifications. Total annual cost for treating psoriasis is estimated to be in the range of $1.6 billion to $4.3 billion dollars [5-7]. Psoriasis is linked with social stigmatization, pain, discomfort, physical disability and psychological distress [8].

### Impact of psoriasis on patients' quality of life

Psoriasis has a significant negative impact on patients' health related quality of life (HRQoL). In a survey by the National Psoriasis Foundation almost 75% of patients believed that psoriasis had moderate to large negative impact on their quality of life (QoL), with alterations in their daily activities [9]. Another study reported that at least 20% of psoriasis patients had contemplated suicide [10]. Furthermore, physical and emotional effects of psoriasis were found to have a significant negative impact at patients' workplace as measured by the validated scales including Work Productivity Assessment Index (WPAI), SF-8, Hospital Anxiety and Depression (HADS) and past medical/psoriasis history [11]. Absenteeism is a greater concern for people suffering from psoriasis than their co-workers without psoriasis with nearly 60% patients reporting missing an average of 26 days a year directly related to their psoriasis [12]. Patients with psoriasis have a higher financial burden due to absenteeism in addition to the cost of caring for their disease [13,14].

Psoriasis patients often experience difficulties like maladaptive coping responses, problems in body image, self esteem, self concept and also have feelings of stigma, shame and embarrassment regarding their appearance [8]. This is often times accompanied by a perception of being evaluated by others based on their disfigurement [8]. Individuals with psoriasis commonly engage in coping strategies to avoid unwanted and unpleasant social consequences. However, most of these strategies fail to improve patients' QoL [14-16]. Discussing their skin condition, covering their lesions, and avoiding contact with people are significantly associated with negative impact on life [11-16]. Studies have indicated that talking to others regarding the non-contagious nature of psoriasis lessens the negative impact on the QoL and thereby reduces social discomfort [17].

Studies that have probed the link between psoriasis and depression hint towards a reciprocal relation between them. Psoriasis patients were more likely to be depressed than the general population with patients' age, education and disease severity being important predictors of psychological distress in the patient cohort [18,19]. Gupta et al. in their study of 127 psoriasis patients found that 9.7% of patients reported their wish to be dead, and 5.5% reported active suicidal ideation at the time of the study [20]. These studies have highlighted the need for psychosocial strategies in treating patients with psoriasis and helping them to improve their overall QoL.

### Determinants of QoL in psoriasis patients

Various factors may be attributed to the lower QoL in psoriasis patients. The chronic and recurring nature of this disease often brings about a feeling of hopelessness in terms of cure for the condition [21]. Patients are constantly concerned with the interference with future plans due to an unexpected outbreak of symptoms. This possibly intensifies due to their lack of control over the disease [21]. Lack of control is one of the most bothersome aspects in psoriasis patients [22].

Many psoriasis patients experience social and psychological difficulties created by their environment [23]. Psoriasis patients may feel humiliated when they need to expose their bodies during swimming, intimate relationships, using public showers, or living in conditions that do not provide appropriate privacy [24]. Many of the patients suffering from psoriasis often feel the need to hide their disease, thus severely affecting their self confidence [25].

People suffering from psoriasis feel that the general public and sometimes their own physicians fail to appreciate the negative impact of psoraisis on their life [10,15]. Psoriasis has an immense impact on social life, with patients frequently complaining of social difficulties and friction with family members [26]. Psoriasis patients frequently feel ashamed and embarrassed about their condition and considered this to be the worst aspect of their disease [27]. High levels of stress in this population may often result from other people reacting to their disease or anticipation of the same [27].

Psoriasis is also associated with limitations in daily activities, occupational, and sexual functioning [14,28,29]. Patients with psoriasis suffer comparable disability as other patients with chronic illnesses [16]. All these factors may have detrimental effect on the patients' QoL [30]. In one qualitative study carried out to assess the determinants of QoL in the US population with psoriasis, body surface area showed the strongest association with decrements in QoL (Spearman's ρ = 0.50; p < 0.0001), among other factors including patients' age, gender, income, duration of psoriasis, and number of physicians seen in last two years. Increasing psoriasis severity was significantly associated with seeking care from multiple physicians and having decrements in income in this population [31]. While measures of body surface area affected by psoriasis are commonly used in clinical trials to assess severity of the disease, there is a movement which argues that QoL standard would be a better method of determining the severity of psoriasis [10]. In the clinic setting, treatment judgments may be largely guided by QoL issues.

### Tools to measure QoL

Various measures have been used to assess QoL in psoriasis patients. These measures may be categorized as psoriasis-specific, skin specific, generic QoL measures, and "mixed" measures. Following section attempts to describe each of these categories with related examples. Psoriasis specific measures are the most sensitive, however the more general measures facilitate comparisons across diseases. Often, studies measuring QoL in psoriasis patients utilize more than one of the available measures mentioned here. Use of these measures varied across different clinical controlled trials examining effect of different pharmacological treatments on QoL of psoriasis patients (Table 1).

**Table 1:** Table Quality of Life Measures and Outcomes in Randomized Controlled Trials for Psoriasis Treatments

**Study Title (Year)**	**Treatment**	**QoL Measures**	**Outcomes**
"Impact of efalizumab on patient-reported outcomes in high-need psoriasis patients: results of the international, randomized, placebo-controlled Phase III Clinical Experience Acquired with Raptiva (CLEAR) trial" (2005) 40	Efalizumab 1 mg/kg/wk (n = 529) or placebo (n = 264) for 12 weeks	1. SF-36 2. DLQI 3. PSA 4. VAS for itching 5. PGPA	QOL, measured using all QOL measures, was significantly higher among the Efalizumab group as compared with the placebo group (p < 0.001)
"Patient-reported outcomes of psoriasis improvement with etanercept therapy: results of a randomized phase III trial" (2005) [41]	Etanercept 50 mg twice weekly (n = 194) Placebo (n = 193), etanercept 50 mg per week (n = 196) during the initial 12-week, double-blind period.	1.DLQI 2.SF-36 3. PGPA	DLQI total score improved by 65–70% in etanercept group compared with 6% in placebo group (P < 0.0001). Significant improvement in etanercept group as measured by other SF-36 and PGPA
"Alefacept in the treatment of psoriasis in patients for whom conventional therapies are inadequate" (2005) [42]	Alefacept (Amevive^®^)	1. PASI 2. DLQI	The QOL effects of alefacept in patients who were not candidates for conventional systemic psoriasis therapies or phototherapy were similar to those reported previously for the overall alefacept-treated population in the phase III studies (p = 0.001).
"Infliximab treatment results in significant improvement in the quality of life of patients with severe psoriasis: a double-blind placebo-controlled trial" (2005) [43]	Intravenous infusions of 3 or 5 mg kg(-1) of infliximab or placebo	DLQI	Infliximab induction therapy resulted in a substantial improvement in HRQOL. At week 10, patients in the infliximab 3- and 5-mg kg(-1) groups showed a median percentage improvement in DLQI scores of 84.0% and 91.0%, respectively, compared with 0% in the placebo group (P < 0.001)
"The efficacy and tolerability of clobetasol propionate foam 0.05% in the treatment of mild to moderate plaque-type psoriasis of nonscalp regions" (2003) [44]	Clobetasol propionate foam (clobetasol foam) 0.05%	PGA	Clobestasol propionate foam 0.05% had greater improvement in QoL as compared to other topical therapies reported by patients.
"Quality of life and clinical outcome in psoriasis patients using intermittent cyclosporine" (2001) [45]	Cyclosporin (Neoral^®^) (n = 255)	1. DLQI 2. PASI	Intermittent short courses of cyclosporin significantly improved the QoL of the patients and decreases the extent and severity of disease and itch
"The impact of a two-compound product containing calcipotriol and betamethasone dipropionate (Daivobet/Dovobet) on the quality of life in patients with psoriasis vulgaris: a randomized controlled trial" (2004) [46]	Combination therapy with topical vitamin D analogue calcipotriol (50 microg g(-1)) and corticosteroid betamethasone dipropionate (0.5 mg g(-1)) vs. calcipotriol monotherapy	1. PDI 2. EuroQoL 5D 3. VAS	Once-daily application of the combination product was found to be superior to calcipotriol twice daily terms of QoL
"The effect of treatment on quality of life in psoriasis patients" (2005) [47]	Treatment with short contact dithranol treatment, UVB phototherapy or inpatient dithranol	1. Dutch short form of the SIP 2. PDI	Comparable improvement in HRQoL with short contact dithranol treatment and UVB phototherapy, inpatients experienced a more impaired HRQoL and showed no significant improvement in HRQoL directly following treatment
"Methotrexate versus cyclosporine in moderate-to-severe chronic plaque psoriasis" (2003) [48]	Methotrexate (n= 44; initial dose, 15 mg per week) or cyclosporine (n= 44; initial dose, 3 mg per kilogram of body weight per day)	1. PASI 2. PGA	The difference in the QOL for both the treatment arms was statistically insignificant
"Calcipotriol vs. tazarotene as combination therapy with narrowband ultraviolet B (311 nm): efficacy in patients with severe psoriasis" (2000) [49]	Combination of UVB (311 nm) and tazarotene vs. UVB (311 nm) plus calcipotriol or vice versa	PASI	No significant differences in QoL of patients in both the regimens
"A comparison of treatment with dithranol and calcipotriol on the clinical severity and quality of life in patients with psoriasis" (1998) [50]	Calcipotriol ointment (50 micrograms/g) twice daily or Dithrocream (short-contact dithranol) 0.1–2%	1. PDI 2. SIP	Significant improvement in patients' QoL as assessed by the PDI and the SIP were seen in both treatment groups, with greater improvement in calcipotriol group

### Psoriasis-specific measures

#### Psoriasis Index of Quality of Life (PSORIQoL)

The PSORIQoL is based on a "needs-based" approach. This instrument is based on the theory that "life gains its quality from the ability and capacity of individuals to satisfy their needs" [30]. This 25 dichotomous item instrument was developed through interviews conducted in three countries in Europe. An American version has also been recently developed. It has the advantage of being based on theory and measuring the impact of the disease on QoL rather than assessing impairment or disability. Moreover, it is expected to work in a uniform manner across patient samples, irrespective of age and gender [30].

#### Psoriasis Life Stress Inventory (PLSI)

The PLSI is a 15-item questionnaire that provides a measure of the daily hassles of psychosocial stress associated with having to cope with everyday events in living with psoriasis. Scores on this scale range from 0 to 45. The PLSI also permits patients to be classified as a function of their distribution of scores into two groups: those patients who react significantly to the stress associated with having psoriasis (score of > 10); and those patients who are not significantly affected with having psoriasis-related stress (score of < 10). The PLSI is scored by having the respondent rate the absolute frequency with which each item has been experienced in the last 4 weeks from (scoring 0) to a great deal (scoring 3) [27].

#### Psoriasis Disability Index (PDI)

The PDI is a 15-item scale that specifically addresses self-reported disability in areas of daily activities, employment, personal relationships, leisure and treatment effects. The items are concerned with the practical effects of psoriasis in every day life [27].

#### Psoriasis Area and Severity Index (PASI) and Simplified PASI (SAPASI)

Four main areas were assessed for calculation of the PASI scores: the head, the trunk, the upper extremities, and the lower extremities, corresponding to 10%,20%,30%, and 40% of the total body area, respectively. The maximum score for PASI is 72. The SAPASI is a self-assessed, using the same criteria as the PASI, but presented in non-professional terminology. Scores on the SAPASI range from 0 to 72 [25]. Though in essence the PASI and the SAPASI are measures for severity of psoriasis, they provide an adequate picture of the impact of the disease on patients' QoL. Studies have indicated an inverse relationship between QoL and severity of psoriasis. Moreover, PASI is the most widely used measure of severity in the research as well as the clinical setting. This makes it an important tool in gauging the impact of the disease on QoL, though other instruments to measure QoL are encouraged. Since PASI or SAPASI do not measure the impact of psoriasis on patients' QoL directly, use of other QoL scales is recommended.

### Skin-specific measures

#### Questionnaire on Experience with Skin Complaints (QES)

The short form of the QES with 23 items is a valid instrument for examination of social and psychic burdens of psoriasis. The recording of stigmatization feeling and of quality of life determines different supplementary aspects of the illness-related stress of patients with chronic skin diseases [32].

#### Dermatology Life Quality Index (DLQI)

The DLQI is a compact self-reported questionnaire to measure HRQoL over the previous week in patients with skin diseases. It consists of 10 items covering symptoms and feelings (items 1 and 2), daily activities (items 3 and 4), leisure (items 5 and 6), work and school (item 7), personal relationships (items 8 and 9) and treatment (item 10). Each item is scored on a four point scale, with higher scores indicating greater impairment in HRQoL [33].

### Generic QOL measures

#### Short Form 36 (SF-36)

The SF-36 health survey is a widely used generic, 36-item, self-reported health status questionnaire assessing 8 domains of health status(1) physical activities;(2) social activities;(3) usual physical role activities;(4) bodily pain;(5) general mental health (6) usual emotional role activities; (7) vitality ;(8)general health perceptions. A score from 0 to 100 is calculated for each subscale, with higher scores indicating better HRQL [25]. The SF-36 may be the best characterized measure for comparing QoL differences across different diseases. The SF-36 was used to show that the impact of psoriasis is as great as that of other major medical disorders [16].

#### Subjective Well Being Scale (SWLS)

The SWLS is a short 5-item instrument designed to measure global life satisfaction. The scale has been validated and correlates with other measures of subjective well-being (SWB). The SWLS was developed to assess satisfaction with the respondent's life as a whole, without assessing satisfaction with specific life event [25].

#### EuroQoL 5D (EQ-5D)

The EQ-5D is a standardized generic instrument developed for describing and valuing health states. The EQ-5D was created for use in population health surveys or in conjunction with a condition-targeted instrument for assessment of outcomes related to specific health conditions or their treatment. It specifically refers to health status at the time of questioning. The first of 2 parts records a patient's health state along 5 dimensions (mobility, self-care, usual activities, pain/discomfort, and anxiety/depression). Each dimension has 3 levels reflecting no problem, some problem, and extreme problem. Respondents are asked to indicate one of the 3 levels along each of the 5 dimensions. This classifies respondents into 1 of 243 distinct health states. The second part of the EQ-5D is a 20-cm VAS that has the end points: "best imaginable health state" (100) and "worst imaginable health state"(0). Respondents are asked to illustrate how they rate their own health stat by drawing a line to that best represents their own health state on that day [25].

### Mixed QoL measures

#### Salford Psoriasis Index (SPI)

The SPI is derived from combining a score of current severity of psoriasis based on the PASI, a score indicating psychosocial disability, and a score based on historical information. The resultant three- figure SPI (signs, psychosocial disability, interventions) is a similar paradigm to the TNM (tumor, nodes, metastasis) classification used for cancer staging [34].

#### Koo-Menter Psoriasis Instrument (KMPI)

The Koo-Menter Psoriasis Instrument (KMPI) is a diagnostic algorithm and a formal measure, to aid in identifying patients with significant impact on QoL warranting systemic therapy. In addition, the KMPI can be used to document and justify treatment decisions for health care payers. While the decision to undertake systemic treatment and the choice of specific treatment plan must ultimately be made mutually by the patient and the physician, these tools are designed to provide information that will be valuable in making informed decisions regarding treatments [35].

### Pharmacological treatments and their impact on QoL

Topical corticosteroids remain the mainstay of psoriasis therapy in the US. Steroid potencies range from class 7 steroids, such as 1% hydrocortisone, which is available in drug- without prescription, to superpotent class 1 corticosteroids such as clobestasol propionate, halobetasol propionate, betamethasone dipropionate [36]. The side effects of topical potent corticosteroids limit their use to an extent, and they are prescribed less frequently outside the US.

Primary treatments for severe psoriasis are phototherapy, systemic retinoids, methotrexate, cyclosporine and newer biological therapies. Ultraviolet B (UVB) phototherapy is an effective treatment for psoriasis and has been the safest way to maintain control of extensive psoriasis over the long-term. If UVB phototherapy is not sufficient to control a patient's psoriasis, then a combination of UVB plus the oral retinoid acitretin is often effective. These therapies often result in prolonged remission of varying duration; however, they are inconvenient for the patient. Advances in our understanding of the immune system in psoriasis have seen the development of biological agents which target molecular and cellular events leading to the disease. Alefacept was approved by the FDA for the treatment of psoriasis in January 2003. It inhibits the activation of T cells and reduces the number of activated memory T cells through apoptosis. As it causes reduction in activated memory T cells, T cells counts are performed to monitor for toxicity. Etanercept which antagonizes Tumor Necrosis Factor (TNF) activity was approved by the FDA in 2002. Studies using infliximab, an anti-TNF anti-body, also have shown efficacy in the treatment of psoriasis. TNF inhibitors are not FDA- approved for psoriasis, however, they are approved for other indications and physicians can prescribe them for psoriasis. Adalimumab is also other TNF inhibitor [37].

Different pharmacological treatments available for psoriasis are found to have varying effects on patients' QoL. Table 1 illustrates outcomes reported in various randomized controlled trials examining the effect of pharmacological intervention on patients QoL. Few studies have reported head to head comparisons of effect of different psoriasis treatments in improving QoL. More recently, studies are focused on understanding the effect of biologic agents on patients QoL, however none of them reported direct comparison of these agents. Clinical trials most often used DLQI or PDI to measure QoL. While very few trials utilized single QoL instrument, others used multiple instruments for QoL measurement. Although the exact reason for utilizing more than one measure for QOL was not mentioned in these studies, this may potentially be done to either compare measures or because no single measure was considered to be superior over the other with each offering a different aspect to the measure of QoL.

### Physicians' role: what we need to know

Counseling patients with psoriasis may improve their mental and psychological condition. Such treatment should be aimed at increasing personal control, encouraging active coping strategies, restructuring negative thoughts about the disease, and encouraging patients to express emotions, seek social support and distract themselves [36]. Inducing remission and achieving reduction in severity of psoriasis (reduce area affected by psoriasis) may not be enough. Pharmacologic interventions should be accompanied by patient education and reassurance by family and social interventions [21].

As in treatment of other medical conditions, establishing a strong physician-patient relationship is the foundation of effective psoriasis treatment. Due to the recurring nature of the disease, patients are not only frustrated with the disease, but also with the care they receive or have received in the past. Physicians have to be empathetic and work with the patient to effectively manage their disease. Establishing this bond and trust between patient and physician will encourage patients to be more complaint to their physician's recommendations concerning treatment and will potentially improve treatment compliance and outcomes [38,39].

The physician may find many opportunities during the patient interview to establish this bond. To start, the physician should sit within touching distance of the patient and palpate the lesions as a part of physical examination. This act helps the patient overcome inhibitions regarding social interactions. Touching communicates that the patient is not untouchable and that the psoriasis does not have to impede intimate social relationships. Physicians should also ask a few leading questions about how psoriasis influences the patient's life. This communicates a sense of empathy and understanding that will assure the patient of the physician's competence in managing psoriasis.

## Conclusion

Psoriasis is a serious condition and is associated with significantly lower QoL. Studies have utilized different measures available to assess QoL of psoriasis patients. Most commonly used measures were psoriasis specific such as PASI and DLQI followed by generic measures such as SF-36. Pharmacological interventions along with patient counseling and education may be an effective strategy to improve QoL among psoriasis patients. Lack of head to head comparisons of available treatment options limits conclusions regarding superiority of one agent over another in improving QoL in psoriasis patients.

## Competing interests

The Center for Dermatology Research is supported by an unrestricted educational grant from Galderma Laboratories. Dr. Feldman has received research, speaking and/or consulting support from Abbott, Amgen, Biogenidec, Centocor, Connetics, Genentech, and Roche.

## Authors' contributions

Ms. Bhosle and Mr. Kulkarni were responsible for the literature review and writing of the manuscript. Dr. Feldman and Dr. Balkrishnan critically revised the manuscript.
